# Rare Cause of Cough-Induced Abdominal Pain

**DOI:** 10.7759/cureus.42530

**Published:** 2023-07-27

**Authors:** Carlos Chaves, Catarina Reigota, Filipe Vilão

**Affiliations:** 1 Gastroenterology, Centro Hospitalar e Universitário de Coimbra, Coimbra, PRT; 2 Internal Medicine, Centro Hospitalar e Universitário de Coimbra, Coimbra, PRT

**Keywords:** cough, rare case report, interests in internal medicine, spontaneous rectus sheath hematoma, acute abdomen

## Abstract

Rectus sheath hematoma is a rare cause of acute abdomen, and its clinical diagnosis is challenging without any imaging methods. We describe a case of a 50-year-old woman with no significant medical history who developed a sudden abdominal pain following a cough paroxysm. On physical examination, there was intense pain after light percussion and tenderness on palpation. Laboratory findings were within normal range. The imaging findings allowed us to diagnose a spontaneous rectus sheath hematoma. A conservative approach was opted. The patient had a favorable course. Accurate diagnosis is of the utmost importance as it may avoid unnecessary interventions, and in some cases, especially for elderly people, it can be fatal. This case alerts us to a rare cause of acute abdomen.

## Introduction

Rectus sheath hematoma is an uncommon condition that can present in a similar fashion to other life-threatening causes of acute abdomen. Awareness of such a condition is vital, as its diagnosis is challenging, in order to reduce iatrogenic procedures. Trauma and antithrombotic medication are the most common risk factors [1]. A thorough medical history and physical examination are fundamental. According to the diagnosis and the patient characteristics (comorbidities and clinical condition), we should decide the best treatment and follow-up. For this reason, we present a case of an infrequent cause of acute abdomen.

## Case presentation

A woman in her 50s presented to the emergency department (ED) due to a viral respiratory infection, being discharged with acetaminophen. A week later, during a cough burst, she developed a sudden, sharp and limitative pain in the left iliac fossa, being admitted to the ED again the following day. The pain was refractory to analgesics, and the patient had no bowel movements since the beginning of the episode. She had no significant medical history except for hypertension and hypothyroidism and her medications did not include antithrombotic drugs.

**Figure 1:**
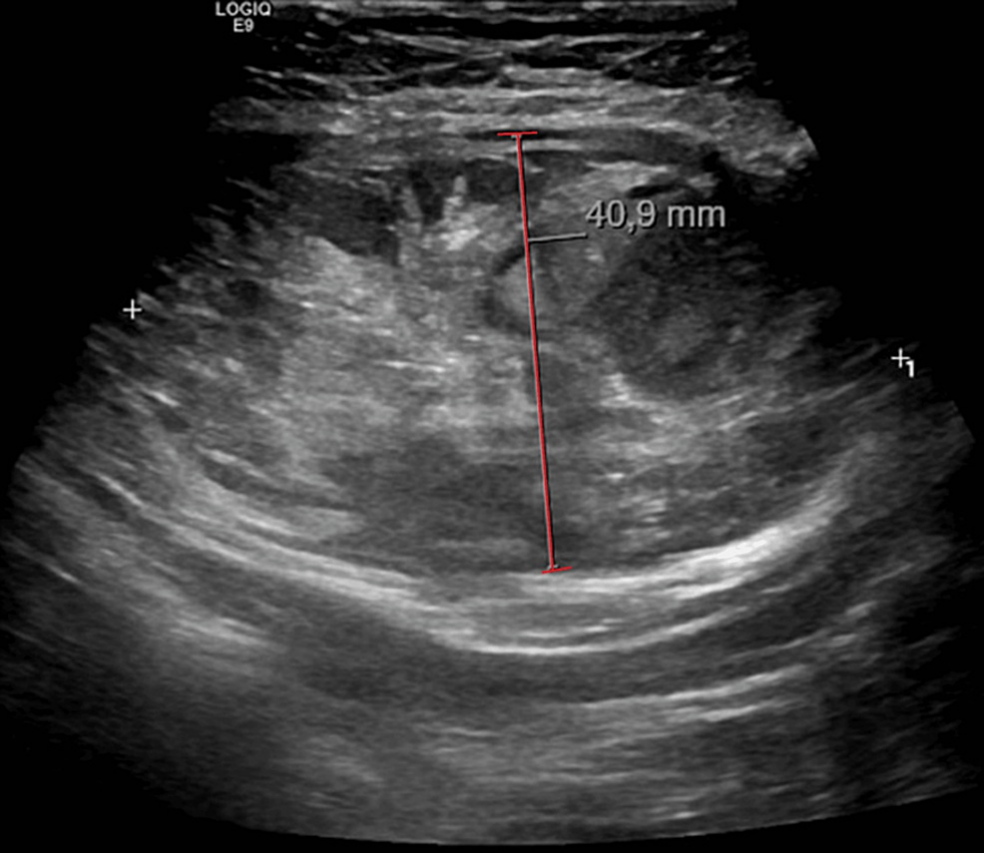
Heterogeneous collection (4x7cm) in the abdominis rectus muscle topography on ultrasound compatible with rectus sheath hematoma.

On examination, although there was intense pain after light abdomen percussion and tenderness on palpation in the left iliac fossa, she had no visible alterations with normal bowel sounds. The blood pressure was 162/95 mmHg with a normal heart rate, no fever or desaturation. The remaining examination had no relevant alterations. Laboratory findings revealed a hemoglobulin of 13.6 g/dL, similar to the previous analysis, leucocytosis and neutrophilia of 18.4 and 13.35x10^9^/L, respectively, normal platelet counts, normal coagulation tests, reactive C protein of 0.54 mg/dL and the remaining lab values were unremarkable. Initially, an abdominopelvic ultrasound was performed and a 4x7cm heterogenic lesion, mainly liquid, in the abdominis rectus muscle topography was seen, with no other abnormalities (Figure 1).

**Figure 2:**
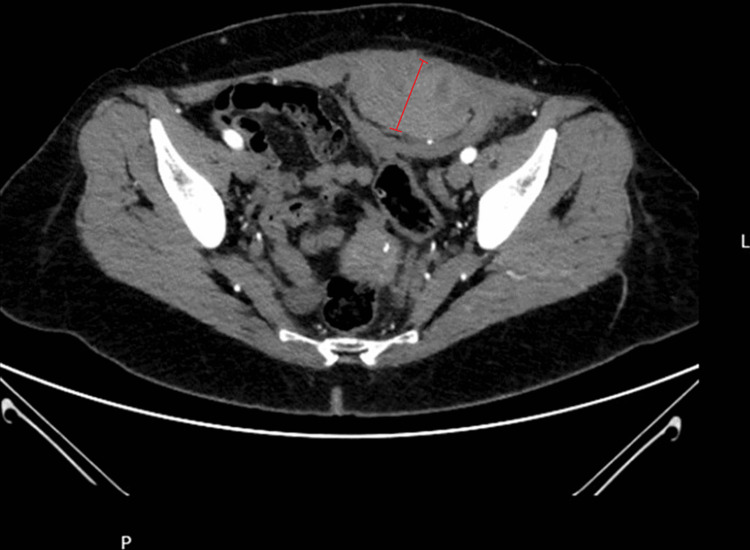
Dense collection within the rectus abdominis sheath, consistent with a hematoma without active hemorrhage after contrast administration.

Subsequently, abdominopelvic CT scan was carried out and showed a large dense collection involving the inferior epigastric artery within the rectus abdominis sheath, consistent with a hematoma without active haemorrhage after contrast administration, measuring 80mm transversally, 48mm anterior-posteriorly and 141mm longitudinally (Figure 2).

Before imaging, potential causes for the signs and symptoms presented by the patient that could cause acute abdomen were considered, namely diverticulitis, acute appendicitis, intestinal obstruction, inguinal hernia strangulation, cystitis, gynaecological pathologies, mesenteric ischemia and so on. Nevertheless, after adequate imaging, the final diagnosis of rectus sheath hematoma was established.

The case was discussed with surgery and interventional radiology colleagues and adoption of conservative measures with ice, rest, analgesics and muscle relaxants was decided, once there was no active hemorrhage. The patient remained under surveillance for 24 hours in the ED. Since there was no significant hemoglobin drop and remained hemodynamically stable, the patient was discharged with the above-mentioned measures.

**Figure 3:**
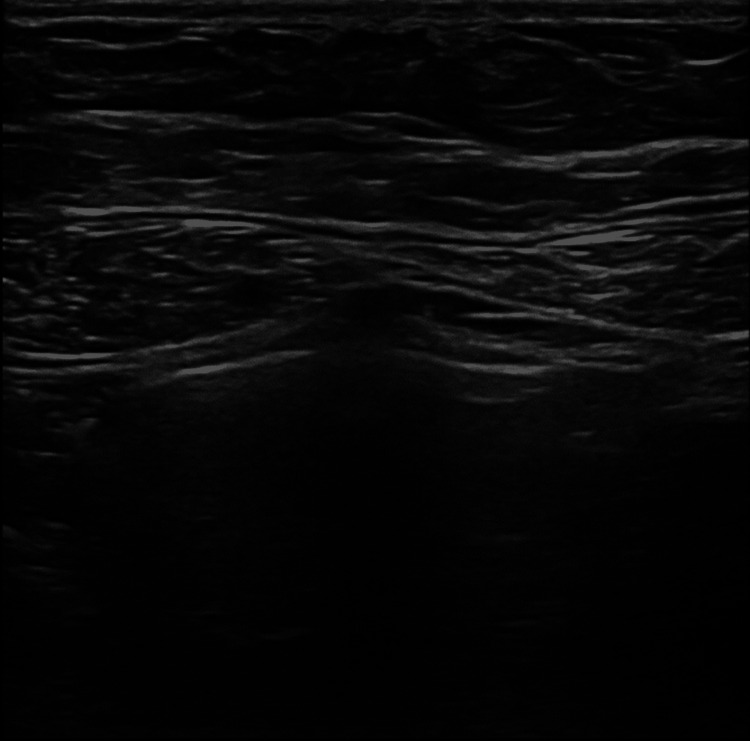
Complete resolution of the hematoma after 12 weeks, with normal findings on ultrasound.

The patient was seen in the outpatient setting two and 12 weeks afterward. There was continuous improvement of abdominal pain with the suggested measures, hemoglobin level remained stable and there was imagological resolution of the hematoma (Figure 3).

## Discussion

Rectus sheath hematoma, whose incidence is unknown [2], is caused due to tearing of superior/inferior epigastric vessels or rupture of the rectus abdominis muscle [3]. Even though classically related to trauma, surgery, pregnancy and antiaggregant/anticoagulant medication [4], we presented a case where such risk factors were not observed. Compression of the inferior epigastric artery against the semicircular line after contraction of the inferior portion of the rectus abdominis muscle may play a role in nontraumatic events [5]. Such cases with spontaneous rupture may happen with strenuous exercise, cough paroxysms, vomiting or during defecation [6]. Usually, this condition presents with acute severe abdominal pain over the ipsilateral inferior quadrant, there may be visible ecchymoses, severe tachycardia and hypotension [5]. In this case, there may be no bowel movements [4,5]. A good history is important, as it may make a clinician consider this diagnosis due to its sudden nature, avoiding unnecessary hospital expenses related to diagnostic tests. Imaging, either with ultrasound or CT scan, is vital to confirm the diagnosis. In uncomplicated cases rectus sheath hematoma can be managed conservatively with rest, ice and analgesia [4]. In complicated cases, when there is hemodynamic instability or organ compression, vascular embolization or hematoma evacuation may be required [4].

## Conclusions

Rectus sheath hematoma can present in a similar fashion to other life-threatening causes of acute abdomen (usually presents with sudden and sharp abdominal pain, accompanied or not by ecchymoses). Spontaneous nontraumatic rectus sheath hematoma may present following strenuous exercise, cough paroxysms, vomiting, or defecation, and requires a high index of suspicion. This case highlights the potential of sustained cough to cause abdominal hematomas even in the absence of anticoagulants and its correct diagnosis is crucial to administer the proper treatment, usually conservative, but in complicated cases, invasive treatment may be considered.
